# Transcranial photobiomodulation improves prefrontal oxygenation and impulse control in adults with ADHD: a randomized controlled trial

**DOI:** 10.1186/s12888-026-08391-5

**Published:** 2026-07-14

**Authors:** Farzad Salehpour, Douglas W. Barrett, Anagh Mirji, Ayla Farzamnia, Vikas Burugu, Nisarg Shah, Francisco Gonzalez-Lima

**Affiliations:** 1 Department of Psychology, Texas Consortium in Behavioral Neuroscience, The University of Texas at Austin, Austin, TX USA; 2 University of Texas Medical Branch, John Sealy School of Medicine, Galveston, TX USA; 3 Department of Psychiatry and Behavioral Sciences, Dell Medical School, The University of Texas at Austin, Austin, TX USA

**Keywords:** Photobiomodulation, Attention-deficit/hyperactivity disorder, Cognitive enhancement, Impulse control, Working memory, Prefrontal oxygenation

## Abstract

**Background:**

Impulsivity in individuals with attention-deficit/hyperactivity disorder (ADHD) diminishes quality of life. Cortical hypometabolism has been hypothesized to contribute to ADHD. Transcranial photobiomodulation (tPBM) is a safe, non-invasive method for stimulating the prefrontal cortex (PFC), yet its cognition-enhancing effect on impulsivity and sustained attention in adults with ADHD remains understudied. This research evaluated whether acute tPBM administration could improve impulsivity and prefrontal oxygenation.

**Methods:**

The current investigation was conducted in two stages. The first stage included 223 participants with and without a history of ADHD, whereas the second stage focused exclusively on 52 adults with ADHD. In the first stage, participants were randomized to receive either active or sham 1064 nm laser tPBM applied to the right PFC, followed by completion of the continuous performance task (CPT). This stage examined the behavioral effects of tPBM on impulsivity and attentional control in adults with ADHD compared with non-ADHD participants. In the second stage, participants completed the CPT before and after the tPBM session. In addition, functional near-infrared spectroscopy (fNIRS) data were acquired before and after tPBM while participants performed a 2-back working memory task, enabling assessment of prefrontal hemodynamic responses.

**Results:**

Behavioral results from the first stage showed that, within the ADHD group, participants receiving active tPBM exhibited a 51% improvement in correct rejections on the CPT compared to sham, reaching levels comparable to non-ADHD participants (*p* = 0.017, large effect size of Cohen’s *d* = 0.93). In the second stage, exploratory analyses suggested that participants receiving ADHD medication showed greater improvements following tPBM compared to non-medicated individuals (*p* = 0.049), which should be interpreted cautiously. Active tPBM also improved correct rejections in the 2-back task (*p* = 0.040). fNIRS analyses demonstrated increased prefrontal oxygenation following active tPBM, with higher oxyhemoglobin (*p* = 0.011) and total hemoglobin (*p* = 0.038) compared to sham. No adverse events were reported. No sex differences were found in the tPBM effects.

**Conclusions:**

These findings suggest that enhancing prefrontal oxygenation via tPBM may strengthen neural networks supporting cognitive functions in adults with ADHD and merit evaluation in repeated-session studies.

**Trial registration:**

ClinicalTrials.gov (ID: NCT05802680) on 03-20-2023 (retrospectively registered). https://clinicaltrials.gov/study/NCT05802680?term=NCT05802680&rank=1.

**Supplementary Information:**

The online version contains supplementary material available at https://doi.org/10.1186/s12888-026-08391-5.

## Introduction

Attention-deficit/hyperactivity disorder (ADHD) is a neurodevelopmental disorder characterized by impulsivity, inattention, and/or hyperactivity [1]. Longitudinal studies have shown that up to 43% of individuals with childhood ADHD continue to experience symptoms into adulthood, highlighting that ADHD is a persistent condition with detrimental effects on quality of life in adulthood [2]. The estimated prevalence of ADHD in adults in the U.S. is 4.25% [3]. Many adults with functional impairment do not receive an ADHD diagnosis or adequate treatment and report a decreased quality of life [4]. Without specific workplace interventions to address inattention, impulsivity, and disorganization problems, adults with ADHD often perform poorly at work [5]. The excess burden costs associated with unemployment and productivity loss in U.S. adults with ADHD were estimated at $95.6 billion, accounting for approximately 78% of the total burden [6]. Even with psychological treatments, ADHD medications provide only limited relief. The non-medical use of prescription stimulant medications also poses significant risks, often including polysubstance misuse [7, 8]. Additionally, stimulant treatment for ADHD in adults may be inappropriate for individuals with prior or current substance use disorders [9]. The scarcity of effective therapies without significant side effects, psychosocial harm, or adherence challenges underscores the need for adjunctive or alternative non-pharmacologic treatments.

Transcranial photobiomodulation (tPBM) is a noninvasive neuromodulation technique that utilizes low-level, non-thermal red to near-infrared (NIR) light (600–1100 nm) to stimulate neural energy metabolism [10]. Recently, the U.S. Food and Drug Administration (FDA) approved PBM for the treatment of age-related macular degeneration [11]. Work over the past decade has demonstrated the potential cognitive-enhancing effects of tPBM in healthy adults as well as those with depression, bipolar disorder, and older individuals with subjective memory complaints [10, 12–15]. These cognitive-boosting effects induced by tPBM are primarily driven by the photo-oxidation of cytochrome *c* oxidase (CCO), the main intracellular photon acceptor for red/NIR light. This process enhances mitochondrial respiration and upregulates the electron transport chain, ultimately supporting increased energy metabolism in the prefrontal cortex (PFC) [16, 17]. In addition to this mitochondrial bioenergetic mechanism, tPBM has been shown to modulate transmembrane transporters and intracellular signaling pathways, including the upregulation of transforming growth factor-beta 1 (TGF-β1) [18]. These other molecular effects lead to downstream processes such as reduced oxidative stress and neuronal apoptosis [19], increased expression of neurotrophic factors including brain-derived neurotrophic factor (BDNF) [20], attenuation of neuroinflammation [21], and modulation of astrocyte and microglial activity toward a neuroprotective phenotype [22]. Recent work has demonstrated that transcranial application of a 1064 nm low-level laser to the forehead upregulated oxidized CCO concentrations and enhanced PFC oxygenation in older euthymic adults with bipolar disorder [23]. These physiological changes were associated with improved speed and accuracy on tasks measuring cognitive flexibility, working memory, and attentional control [13].

A shared pathophysiology of ADHD and bipolar disorder involves mitochondrial dysfunction and prefrontal hemodynamic dysregulation, along with impairments in the central executive network, dorsal attention network, and fronto-striatal circuitry [24, 25]. Impulsivity and poor behavioral inhibition and inattention are common deficits in individuals with bipolar disorder and ADHD [26]. A functional near-infrared spectroscopy (fNIRS) study in adults with ADHD has shown reduced prefrontal hemodynamic response during a response inhibition task [27]. A meta-analysis focusing on right hemisphere activation revealed ADHD-related hypoactivation in the right ventrolateral PFC (vlPFC) and other brain regions during response inhibition tasks, while hypoactivation in the right dorsolateral PFC (dlPFC) was observed during attention tasks [28]. Consistent with these lateralized findings, prior tPBM studies targeting attention and executive function have predominantly applied stimulation over the right PFC, suggesting a preferential role of this region in modulating cognitive control processes [12, 29].

Despite growing interest in tPBM as a potential intervention for ADHD, relatively few studies have investigated its effects in this population, including one double-blind, randomized, sham-controlled trial in children with ADHD [29] and an open-label non-randomized study in adults with ADHD [30]. However, controlled investigations examining the acute cognitive and hemodynamic effects of tPBM in adults with ADHD remain scarce. Therefore, the present study evaluated the acute effects of 1064 nm tPBM applied to the right PFC on cognitive performance and prefrontal hemodynamics in adults with ADHD using a single-blind, randomized, sham-controlled design. The current investigation was conducted in two stages. The first stage examined the behavioral effects of tPBM on impulsivity and attentional control in adults with ADHD compared with non-ADHD participants. The second stage investigated the underlying prefrontal hemodynamic mechanisms of tPBM-related cognitive enhancement using fNIRS, assessing changes in oxygenated hemoglobin (Δ[HbO]) and total hemoglobin (Δ[HbT]) concentrations. This two-stage design allowed for the examination of both behavioral effects in a mixed ADHD/non-ADHD sample and mechanistic effects in a clinically diagnosed ADHD cohort.

## Materials and methods

### Participants and experimental designs

This study was conducted in the Department of Psychology at The University of Texas at Austin. All participants were explicitly informed that they could withdraw consent and discontinue participation at any time without consequence. After verbally affirming their full understanding and willingness to participate, all participants signed a written informed consent form. Then, participants provided demographic and educational information, as well as details on their medical and psychiatric history, and current medications. Afterward, they completed the paper-based Adult ADHD Self-Report Scale (ASRS-v1.1) screening questionnaire [31]. ADHD case ascertainment was based on participants’ self-reported history of a prior medical diagnosis in combination with ASRS psychometric screening. No structured clinical interviews (e.g., MINI or SCID) or formal verification of medical records were conducted. Comorbid psychiatric conditions were not systematically assessed beyond self-reported medical history. All participants were blind to group assignment and were informed that they would be assigned to either active tPBM or sham control groups.

**Fig. 1:**
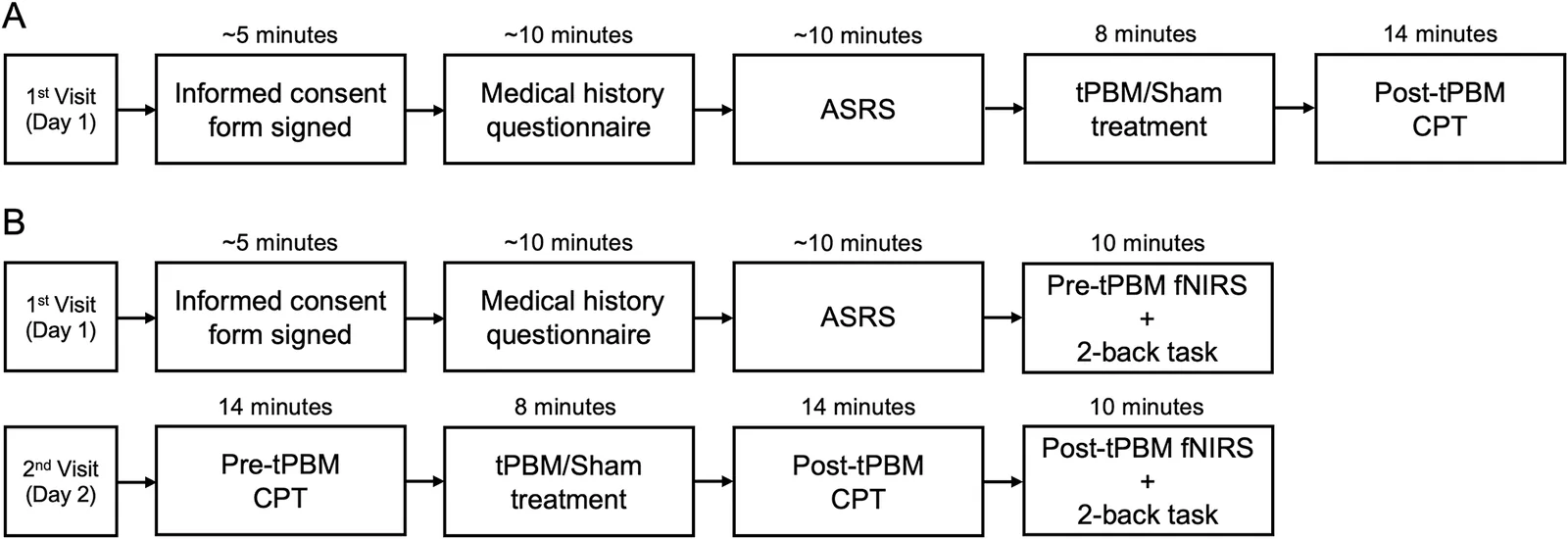
Schematic representation of experimental procedures and timeline for (**A**) stage 1 and (**B**) stage 2

In the initial behavioral stage of this investigation, 223 participants were randomly assigned using simple randomization by the first author to one of four groups: (1) non-ADHD receiving sham tPBM, (2) non-ADHD receiving active tPBM, (3) ADHD receiving sham tPBM, or (4) ADHD receiving active tPBM. Randomization was concealed by assigning a number to each participant. The personnel who enrolled and those who assigned participants to the interventions did not have access to the random allocation sequence. Each participant underwent either sham or active tPBM for eight minutes. Immediately afterward, they completed the Conners’ CPT [32] (Fig. 1A). In the second stage of this investigation involving fNIRS hemodynamics, 52 participants with ADHD were randomized into two groups to receive either sham or active tPBM. On Day 1, participants first completed a pre-fNIRS assessment, during which they performed a 2-back task. On Day 2, approximately 24 h later, they completed the pre-CPT, followed by either sham or active tPBM for eight minutes. Immediately afterward, they completed the post-CPT and then underwent a post-fNIRS assessment, again performing the 2-back task (Fig. 1B). The investigators conducted the pre-fNIRS session and simultaneous 2-back task on Day 1 and scheduled the remaining procedures for Day 2 to minimize cumulative cognitive load within a single session and reduce potential fatigue-related confounds.

Upon completion of all study procedures, a follow-up email was systematically sent, requesting all participants to report any side effects or adverse events they may have experienced during or after the session. None of the participants reported adverse effects or harms.

### Inclusion and exclusion criteria

All participants had to be 18 years or older. For the first stage of the investigation, participants included individuals with and without a history of medically diagnosed ADHD, either on stable ADHD medication (with no changes within two weeks prior to the study) or not taking medication. For the second stage of the investigation, only individuals with a history of medically diagnosed ADHD were eligible, following the same medication criteria. ADHD diagnoses encompassed all three presentations (predominantly inattentive, predominantly hyperactive-impulsive, and combined). Participants were instructed not to alter their use of prescribed psychotropic medications. Participants who were pregnant or had received sham or active tPBM within the past five weeks were excluded.

### Sample population details

**Fig. 2:**
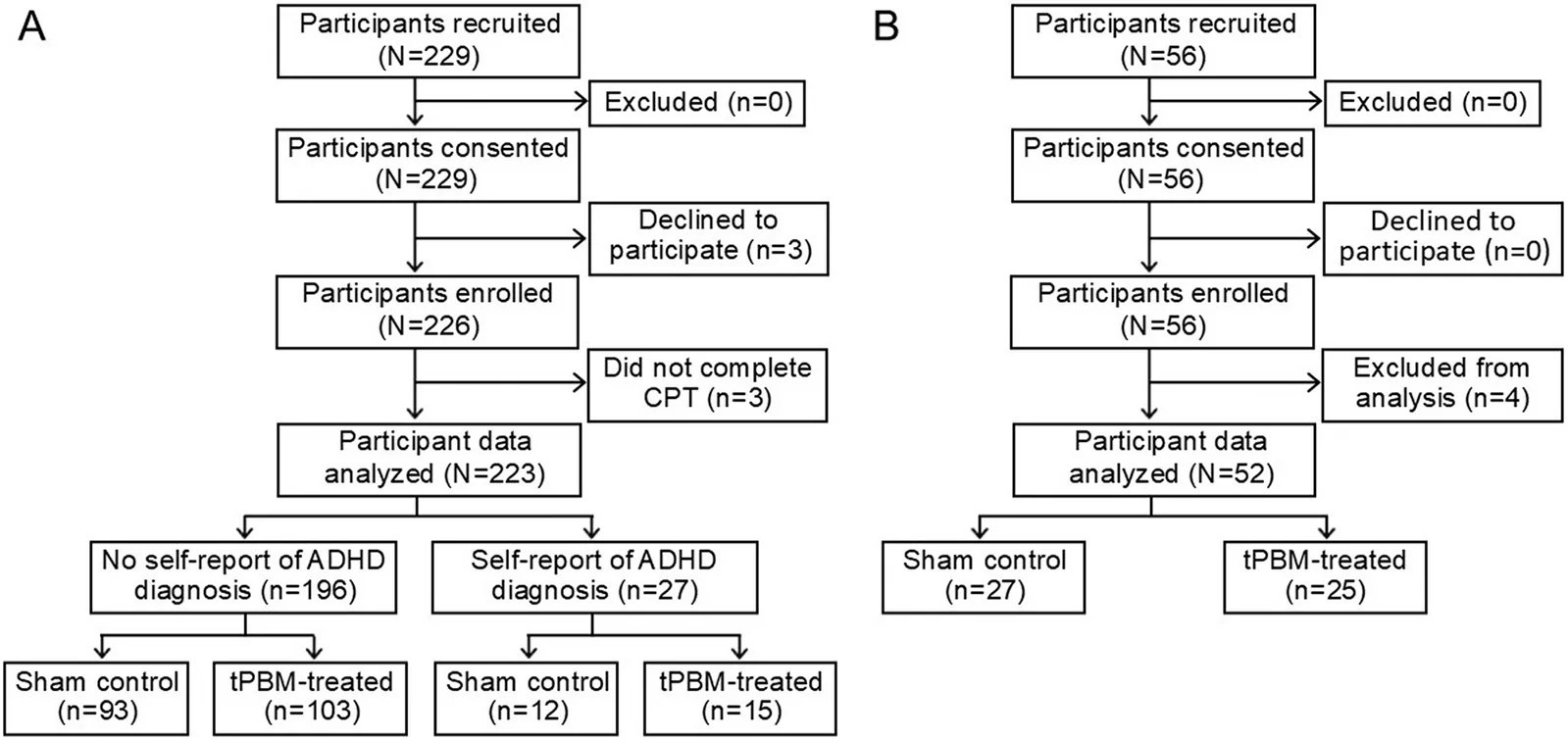
CONSORT diagram of participant enrollment and group assignment in (**A**) stage 1 and (**B**) stage 2. Abbreviations: ADHD, attention-deficit/hyperactivity disorder; CPT, continuous performance task; tPBM, transcranial photobiomodulation

Figure 2 depicts CONSORT diagrams that outline participant enrollment, inclusion, and exclusion for first stage (Fig. 2A) and second stage (Fig. 2B) of the investigation. In the first stage, participants consisted of 155 women and 68 men, with a mean age of 19.5 (standard deviation [SD] = 1.95, range = 18–38 years). In the second stage, participants consisted of 35 women and 17 men, with a mean age of 23.35 (SD = 9.4, range = 18–61 years) (Table 1).

**Table 1:** Participant demographics by sex, age, and ADHD medication status for each group

	Non-ADHD Sham tPBM	Non-ADHD Active tPBM	ADHD Sham tPBM	ADHD Active tPBM
Stage 1				
Participants (n)	93	103	12	15
Sex (M/F)	25/68	31/72	7/5	5/10
Age (mean ± SEM)	19.3 ± 0.13	19.3 ± 0.14	20.5 ± 0.39	21.5 ± 1.34
On ADHD Medications (n)	1	2	5	8
Stage 2				
Participants (n)	-	-	27	25
Sex (M/F)	-	-	10/17	7/18
Age (mean ± SEM)	-	-	24.15 ± 2.06	22.48 ± 1.57
On ADHD Medications (n)	-	-	16	11

Abbreviations: ADHD, attention-deficit/hyperactivity disorder; tPBM, transcranial photobiomodulation; SEM, standard error of the mean

Participants were English-speaking adults of any gender and ethnic background. Individuals with and without a history of medically diagnosed ADHD were recruited through an online participant pool management tool. Additional participants were recruited from the community and offered $10 compensation. Of the 229 recruited for the first stage of the investigation, 223 were included in the final analysis. Two participants declined to participate after arrival at their appointment. Two participants received tPBM and asked to end the study visit without completing the CPT for personal reasons unrelated to the study. Two participants received sham treatment, but did not make any keypresses in response to any letter stimuli during the CPT. For the first stage of the study, participant recruitment occurred between February 2022 and December 2022.

Although the first behavioral stage of the investigation yielded a large effect size (Cohen’s d = 0.93) based on CPT performance, the investigators selected a more conservative estimate to ensure statistical rigor and broader generalizability for the second stage of the investigation involving fNIRS. Based on a power analysis with an alpha level of 0.05, power of 0.80, and a conservative effect size estimate of Cohen’s d = 0.80, the investigators aimed to recruit 30 participants per group to achieve a final sample of 26 participants per group, accounting for an anticipated 15% attrition rate (*n* = 4). Of the 56 participants recruited, 52 were included in the final analysis. Four participants were excluded: one due to inconsistencies in eyeglass use between visits; another because their session was discontinued due to improper calibration of the laser device; a third participant did not return for their second visit; and the fourth participant performed the 2-back task incorrectly, as the experimenter confirmed at the end of the session. For the second stage of the study, participant recruitment occurred between August 2023 to June 2024.

### Medications

A complete list of individual medications for the two stages of this investigation is provided in two Supplementary Tables (S1 and S2). Since some participants were taking more than one medication, the total numbers of medication usage reported in the tables was greater than the total number of subjects.

Among the 223 participants in the first stage of the investigation, 178 (79.8%) reported taking no psychotropic medications. In the active tPBM group, 23 of 118 participants reported taking psychotropics. In the sham group, 22 of 105 participants reported taking psychotropics. Among the 45 participants who reported using psychotropic medications, the most common classes included selective serotonin reuptake inhibitors (SSRIs) (*n* = 27), stimulants (*n* = 16), other antidepressants (*n* = 8), and anxiolytics/sedatives (*n* = 7). One participant reported taking the opioid antagonist naltrexone. Another participant reported taking mood stabilizers lithium carbonate and lamotrigine.

Of the 52 participants in the second stage of the investigation, 25 (48.1%) reported current use of psychotropic medications. These included stimulants (*n* = 23), antidepressants (*n* = 14), and other psychotropic agents (*n* = 18), such as anxiolytics, antipsychotics, and mood stabilizers. Additionally, non-psychotropic medications were reported by 20 participants.

A chi-square test of independence was conducted to examine the relationship between treatment group and psychotropic medication status. Participant randomization of group assignment did not result in any significant differences in the frequency of participants reporting psychotropic medication use between the sham and active tPBM groups (stage 1: *p* = 0.269; stage 2: *p* = 0.271).

### Adult ADHD self-report scale (ASRS-v1.1)

Participants reported symptom ratings using the ASRS 18-item screening checklist assessing ADHD symptom dimensions of inattention (9 items) and hyperactivity-impulsivity (9 items). Each item was rated on a scale from 1 (“Never”) to 5 (“Very Often”) for each symptom over the past six months [31]. Scores were calculated by tallying all answers for questions 1–6 in Part A of the checklist. Participants who check marked four or more of these questions were scored as positive for ADHD symptoms. Cumulative ASRS scores were also calculated for each participant. For all 18 questions in Parts A and B, responses were assigned values from 1 to 5: “Never” = 1, “Rarely” = 2, “Sometimes” = 3, “Often” = 4, and “Very Often” = 5. The total cumulative ASRS score ranged from 18 to 90, with higher scores approaching 90 reflecting greater severity of ADHD symptoms.

### tPBM treatment procedure

**Fig. 3:**
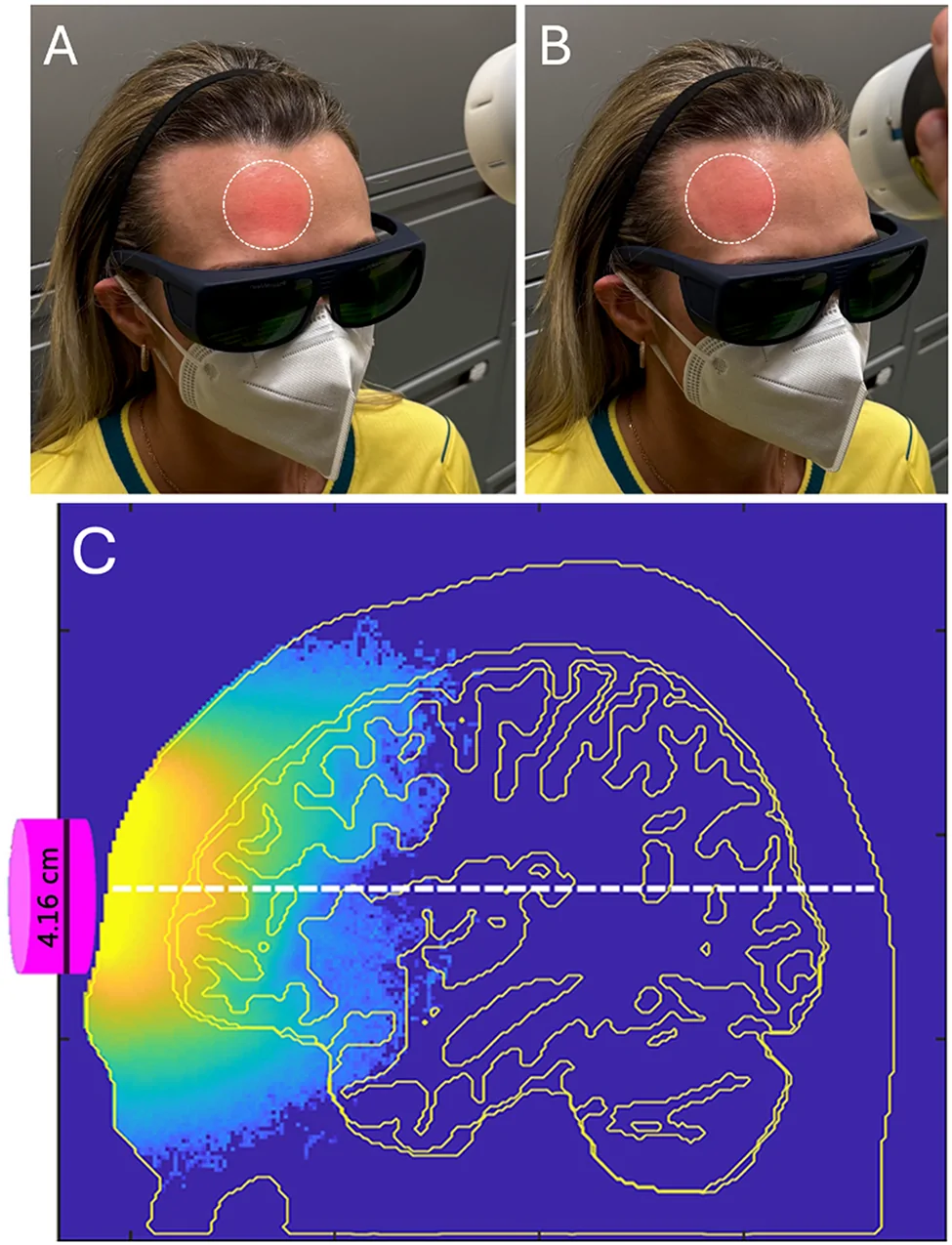
The HD Laser, with a collimated aperture diameter of 4.16 cm, was used to administer tPBM. Stimulation regions over the right forehead included (**A**) a medial area extending from Fp2 and (**B**) a lateral area extending from Fp2, effectively covering the right frontopolar PFC and the right ventrolateral PFC, respectively. The individual shown in the photographs is a volunteer model who provided written informed consent for publication of the images and is not a participant in the study. (**C**) Monte Carlo simulation of 1064 nm light distribution in a sagittal view of the head, illustrating its penetration profile (yellow 5%, orange 2%, light blue < 1%) [33]

The tPBM was administered using a continuous-wave, 1064-nm wavelength laser (HD Laser, Cell Gen Therapeutics LLC, Dallas, TX). The laser is well collimated with a 4.16 cm diameter aperture. A single session tPBM was performed. The tPBM session lasted eight minutes, consisting of eight one-minute treatments alternating between two sites on the right forehead, medial and lateral to the EEG Fp2 point (Fig. 3A and B). With an output power of 3.4 W, the laser irradiance was 250 mW/cm² (3400 mW/13.6 cm² = 250 mW/cm²), and the fluence at each irradiation site was 60 J/cm² (250 mW/cm² × 240 s = 60 J/cm²). All participants and researchers wore protective eyewear during laser operation, and participants were instructed to keep their eyes closed throughout the tPBM treatment. Previous studies have demonstrated that approximately 2% of this 1064 nm light penetrates the supraorbital frontal cranium in post-mortem human tissue [12], and computational modeling has further shown that 1064 nm light exhibits greater penetration through the forehead compared to 660 nm and 810 nm wavelengths [33] (Fig. 3C).

Sham tPBM was delivered at the same sites and for the same duration as active tPBM, using the same device but without emitting the invisible 1064-nm light. To ensure blinding, the laser device produced the same sound regardless of whether it was programmed in active or sham mode. The sensation experienced by participants was comparable in both conditions, as the heat produced by the device was minimal and not perceptible. To assess the effectiveness of blinding, participants were asked at the end of the second stage whether they believed they had received active or sham tPBM (*n* = 52; an “unsure” response option was available, but no participants selected this option), and 58% correctly identified their assigned condition, while 42% provided an incorrect response. A chi-square goodness-of-fit test comparing these responses to chance levels revealed no significant difference (χ²(1) = 1.23, *p* = 0.267), suggesting that participants were not able to reliably distinguish between the sham and active tPBM conditions, indicating effective blinding. All interventions and comparator sham treatments were delivered as intended.

### Conners’ continuous performance task (CPT)

Participants completed a computer-based Conners’ CPT to assess sustained attention and response control [32]. The task was implemented using the Psychology Experiment Building Language (PEBL), an open-source software for cognitive testing. Each CPT session consisted of 360 trials over 14 min. Participants were instructed to press the spacebar as quickly as possible in response to target letters, which included A, B, C, D, E, F, G, H, I, J, L, M, O, P, Q, R, S, and U, but to withhold responses when the letter X appeared. The primary outcome variable was the number of correct rejections: instances where participants successfully inhibited pressing the spacebar when the letter X appeared.

### 2-back task

A 2-back visual working memory task lasted for 10 min both before and after the sham or active tPBM session, while simultaneously undergoing fNIRS. The task began with a 5-minute “rest phase”, during which participants were instructed to relax while maintaining focus on a central fixation cross. This was followed by a 5-minute “2-back phase”, during which individual letters were presented sequentially at the center of a computer screen. Participants were instructed to click on the bottom left corner of the trackpad (equivalent to a left-click) whenever the current letter matched the one presented two trials earlier. They were asked to respond as quickly and accurately as possible [34]. With fNIRS, the 2-back task was used to engage and activate the PFC, providing a cognitive challenge that elicited measurable hemodynamic responses. By including a rest and an active phase, this task enabled the investigators to examine tPBM effects under two distinct cognitive modes: rest, associated with the default mode network, and active task engagement, associated with the frontoparietal attention network [35].

### Acquisition and processing of fNIRS data

fNIRS was used to measure cerebral oxygenation in PFC regions. Participants wore a head-mounted device (NIRSIT device, Soterix Medical Inc., Woodbridge, NJ) before and after tPBM treatment to assess changes in concentration of oxygenated hemoglobin (Δ[HbO]) and total (oxygenated plus deoxygenated) hemoglobin (Δ[HbT]) that may have resulted from tPBM. Data was processed using the NIRSIT Analysis Tool provided by (OBELAB Inc., Seoul, South Korea) using standard procedures as reported in previous spectroscopy studies of tPBM effects on brain hemodynamics in the PFC [13]. The data was subjected to a low-pass filter of 0.1 Hz to remove noise from sources unrelated to the signal such as respiration and cardiovascular artifacts. The average of the first five seconds of spectroscopic measurements provided the baseline light intensity (I_0_) for the modified Beer-Lambert Law, which is a formula used to convert light intensity changes to changes in concentration of oxygenated/deoxygenated hemoglobin for near-infrared spectroscopy data [36]. Any channel identified with a signal-to-noise ratio below 25 was rejected. Δ[HbO] and Δ[HbT] in the 48-channel NIRSIT montage were averaged across each 10-minute fNIRS session to create an “all-channels” index of concentration changes for these variables during both the pre-tPBM and post-tPBM fNIRS sessions. A difference score (post-tPBM session minus pre-tPBM session) was then calculated using each individual participant Δ[HbO] and Δ[HbT] to standardize the post-tPBM concentration changes across individuals.

### Statistical analysis

All data were expressed as mean ± SEM, with significance defined as *p* < 0.05. Missing data was not included in the analysis, as reflected by the degrees of freedom in each analysis. No unplanned analyses were done. The statistical software Jamovi was used. The primary behavioral outcome measure, or dependent variable, was the total number of correct rejections during the CPT. The variable “diagnosis” was defined as whether participants had a diagnosis of ADHD on the medical questionnaire. The variable “treatment” was defined as whether participants received sham or active tPBM. In the first stage data analysis, both “diagnosis” and “treatment” were used as between-subject variables in a 2 × 2 analysis of variance (ANOVA). Post-hoc comparisons were performed using one-way ANOVAs. Linear regressions assessed the relationship between total cumulative ASRS score and CPT total correct rejections.

In the second stage analysis, a three-way repeated-measures ANOVA was conducted with treatment group (sham tPBM vs. active tPBM), ADHD medication status (medicated vs. non-medicated), and phase (pre-tPBM vs. post-tPBM) as factors. Additional analyses included one-way and two-way ANOVAs to evaluate interactions among treatment, phase, and medication status. Non-parametric Mann–Whitney U tests were used when assumptions of normality or homogeneity were violated. fNIRS data were analyzed using Welch’s t-tests and Mann–Whitney U tests to examine group PFC differences in Δ[HbO] and Δ[HbT] concentrations.

## Results

### Stage 1: Effects of tPBM on impulsivity in adults with ADHD compared with non-ADHD participants

#### Effects of tPBM treatment and ADHD diagnosis on total correct rejections in CPT

**Fig. 4:**
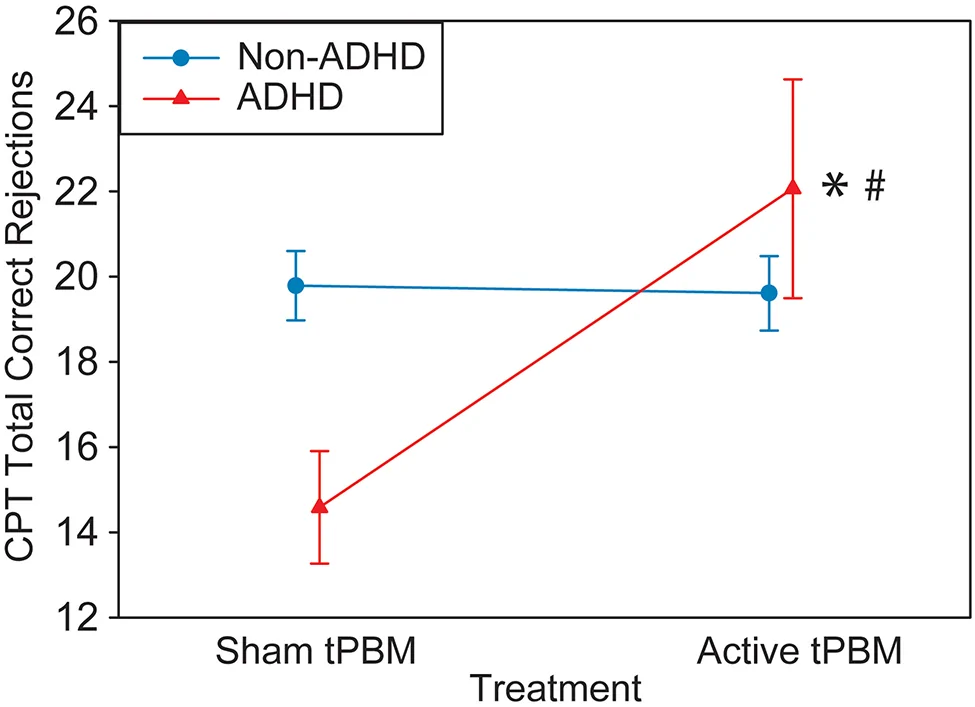
CPT performance (total number of correct rejections) in the ADHD and non-ADHD groups after sham or active tPBM treatment. Within the ADHD group, active tPBM significantly improved performance compared to sham tPBM (*p* = 0.017, Cohen’s *d* = 0.93). ^*^Significant main effect of treatment, *p* < 0.05; ^#^Significant interaction of treatment by diagnosis, *p* < 0.05. Data are presented as mean ± standard error of the mean (SEM)

The first stage of the investigation revealed a significant main effect of tPBM treatment (F_(1,219)_ = 4.488, *p* = 0.035) and a significant diagnosis × treatment interaction (F_(1,219)_ = 4.924, *p* = 0.028) (Fig. 4). Post-hoc comparisons confirmed that within the non-ADHD group, the effect of tPBM treatment was not significant (F_(1,194)_ = 0.021, *p* = 0.885), while within the ADHD group, the effect of tPBM treatment was significant (F_(1,25)_ = 6.71, *p* = 0.017), with a large effect size of Cohen’s *d* = 0.93. Within the ADHD group, those treated with tPBM showed a 51% improvement in total correct rejections over the sham group. CPT performance in the ADHD group after active tPBM treatment was comparable to that of the non-ADHD group.

A post-hoc comparison revealed that among participants who received active treatment, the ADHD group had significantly fewer correct rejections than those without ADHD (F_(1,103)_ = 5.02, *p* = 0.027). Among participants who received sham treatment, the ADHD group exhibited 26% fewer correct rejections than the non-ADHD group, corresponding to a moderate-to-large effect size of Cohen’s *d* = 0.687. These findings indicate that a self-reported history of medically diagnosed ADHD effectively identified a subgroup with significantly lower CPT performance in the primary outcome measure.

After Bonferroni correction for these post-hoc comparisons, the large tPBM effects remained statistically significant at *p* < 0.05. There was no significant interaction effect involving sex when included as a covariate in the analysis (*p* > 0.05).

#### Association between ASRS scores and total correct rejections in the CPT among ADHD and non-ADHD participants

**Fig. 5:**
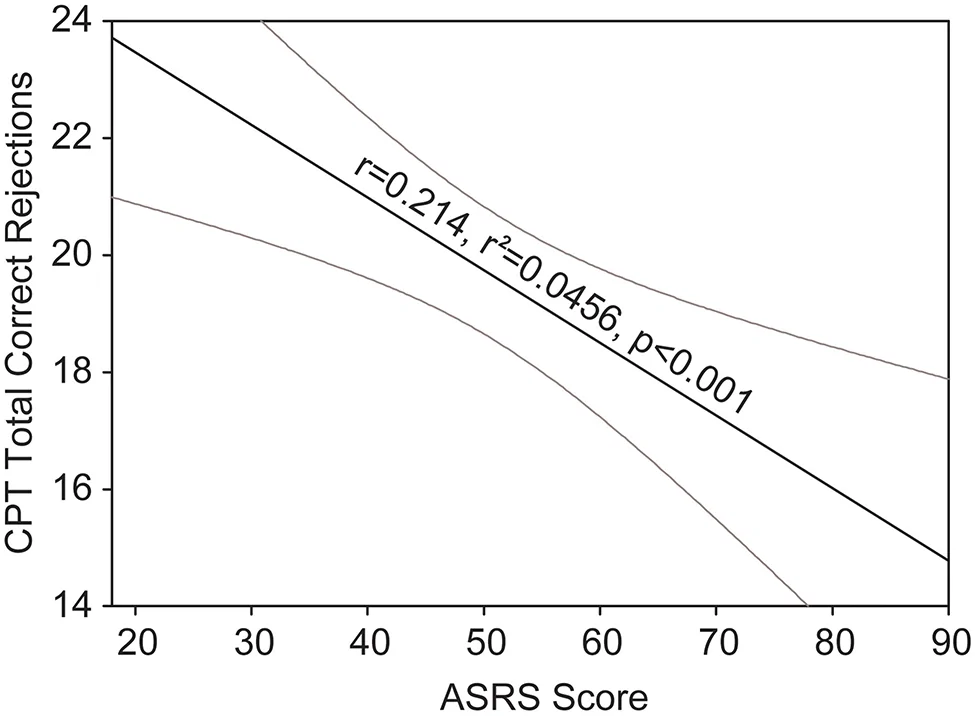
Significant inverse correlation between CPT total correct rejections and ASRS scores across all participants (*r* = -0.214, r² = 0.0456, *p* < 0.001). Higher ASRS scores were associated with poorer CPT performance, indicating greater impulsivity. Depicted is the best-fit line, as well as 95% confidence intervals (above and below the best-fit line)

Regression analyses were conducted to examine the relationship between ASRS scores and total correct rejections across all participants (Fig. 5). ASRS scores were significantly inversely correlated with total correct rejections, indicating that higher ASRS scores predicted poorer CPT performance and greater impulsivity (Pearson’s *r* = -0.214, r^2^ = 0.0456, *p* < 0.001). A similar significant inverse correlation was observed within both the sham and active tPBM-treated ADHD groups (sham tPBM: Pearson’s *r* = -0.229, r^2^ = 0.0526, *p* = 0.019; active tPBM: Pearson’s *r* = -0.205, r^2^ = 0.0419, *p* = 0.026).

**Fig. 6:**
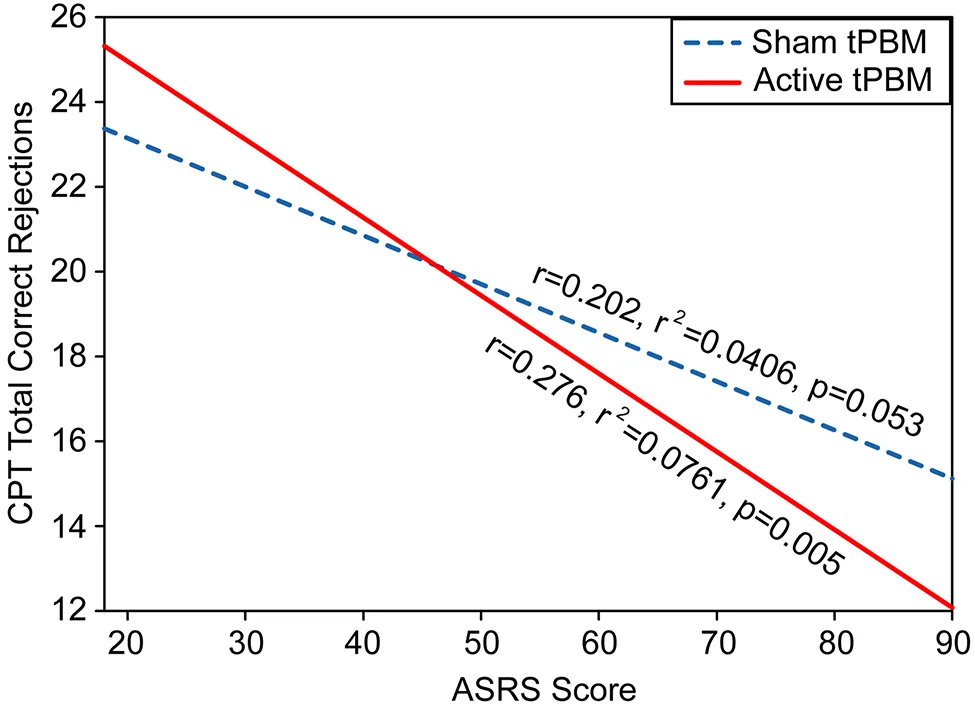
Inverse association between ASRS scores and CPT total correct rejections in the non-ADHD group. The correlation was statistically significant in the active tPBM group (*r* = -0.276, r² = 0.0761, *p* = 0.005), but only approached significance in the sham tPBM group (*r* = -0.202, r² = 0.0406, *p* = 0.053). Higher ASRS scores were associated with poorer CPT performance in both groups. Best-fit lines are shown for both the sham tPBM-treated and active tPBM-treated subgroups

To examine the relationship between ASRS scores and CPT total correct rejections in the non-ADHD group, regression analyses were conducted separately for sham and active tPBM-treated participants (Fig. 6). A Fisher r-to-z transformation test confirmed that the two correlation coefficients did not significantly differ (test statistic Z = 0.54; *p* = 0.5892). Additionally, a one-way ANOVA using a median split of ASRS scores within the non-ADHD group revealed that individuals with higher ASRS scores had significantly fewer total correct rejections than those with lower ASRS scores (F_(1,194)_ = 7.70, *p* = 0.006).

#### Non-Significant Interaction of tPBM Treatment and ADHD Medication Status on Total Correct Rejections in CPT

The main effect of medication was not significant (F_(1, 23)_ = 0.017, *p* = 0.897), suggesting no difference in performance between medicated and non-medicated participants in this small sample of adults with ADHD. Moreover, the interaction between tPBM treatment and medication status was not significant (F_(1, 23)_ = 0.429, *p* = 0.521).

Similarly, to examine the effect of any psychotropic medication use on tPBM treatment response, another one-way ANOVA was conducted within all the active tPBM-treated group, with psychotropic medication usage (present vs. absent) as the independent variable and total correct rejections as the dependent variable. This analysis also showed no significant effect of psychotropic medication use on total correct rejections (F_(1,116)_ = 1.27, *p* = 0.269).

### Stage 2: Cognitive tPBM effects and PFC hemodynamics in adults with ADHD

#### Exploratory interaction of pre-/post-tPBM phase, tPBM treatment, and medication status on total correct rejections in the CPT in adults with ADHD

**Fig. 7:**
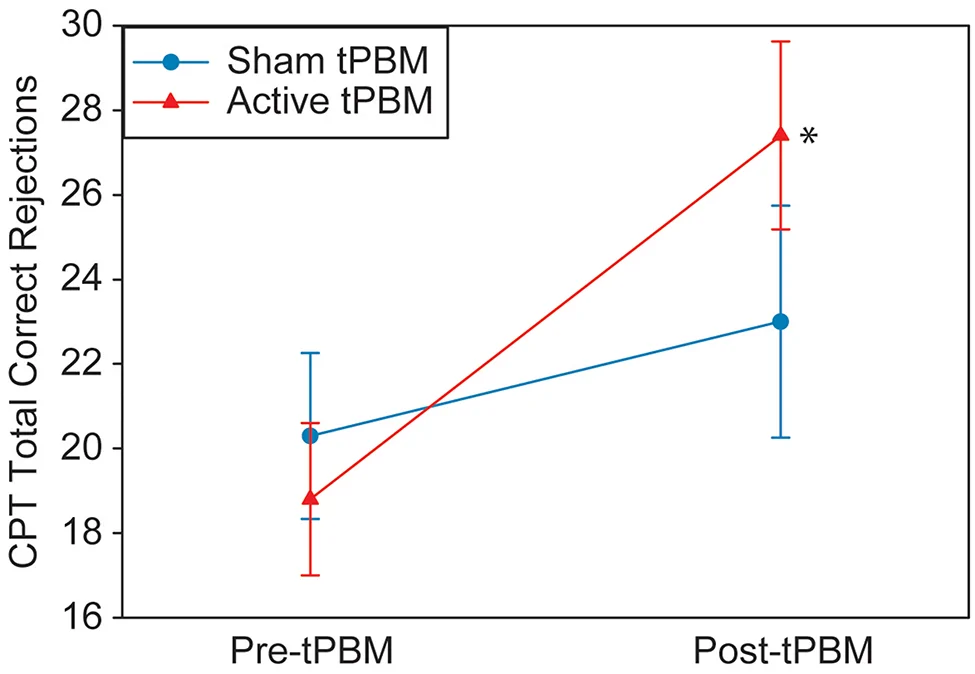
Total number of correct rejection trials in the CPT for the medicated group of adults with ADHD, before and after receiving either sham or active tPBM. A significant three-way interaction was found between phase (pre-/post-treatment), tPBM treatment, and ADHD medication status (*p* = 0.049, η²*p* = 0.083). This result indicates that the greatest improvement in impulse control was observed in participants who were taking ADHD medication and received active tPBM. Significant main effects were also observed for phase and for the phase × medication status interaction. Data are presented as mean ± SEM. *p < 0.05

In the initial analysis for the second stage of the investigation, pre- to post-treatment changes in CPT performance were compared between the active tPBM and sham groups in adults with ADHD, and no significant treatment effect was observed. Given this, and considering the potential influence of clinical variables, an exploratory analysis was conducted to examine whether ADHD medication status moderated treatment-related changes using a three-way interaction model. This analysis revealed a significant phase × treatment × medication status interaction on total correct rejections (F_(1, 45)_ = 4.057, *p* = 0.049, η²*p* = 0.083), indicating a moderate effect size. Significant main effects of phase (F_(1, 45)_ = 6.313, *p* = 0.016, η²*p* = 0.123) and a phase × medication status interaction (F_(1, 45)_ = 5.285, *p* = 0.026, η²*p* = 0.105) were also observed. These findings suggest that treatment-related changes may differ as a function of medication status; however, given the exploratory nature of this analysis, they should be interpreted with caution (Fig. 7). There was no significant interaction effect involving sex when included as a covariate in the analysis (*p* > 0.05).

**Fig. 8:**
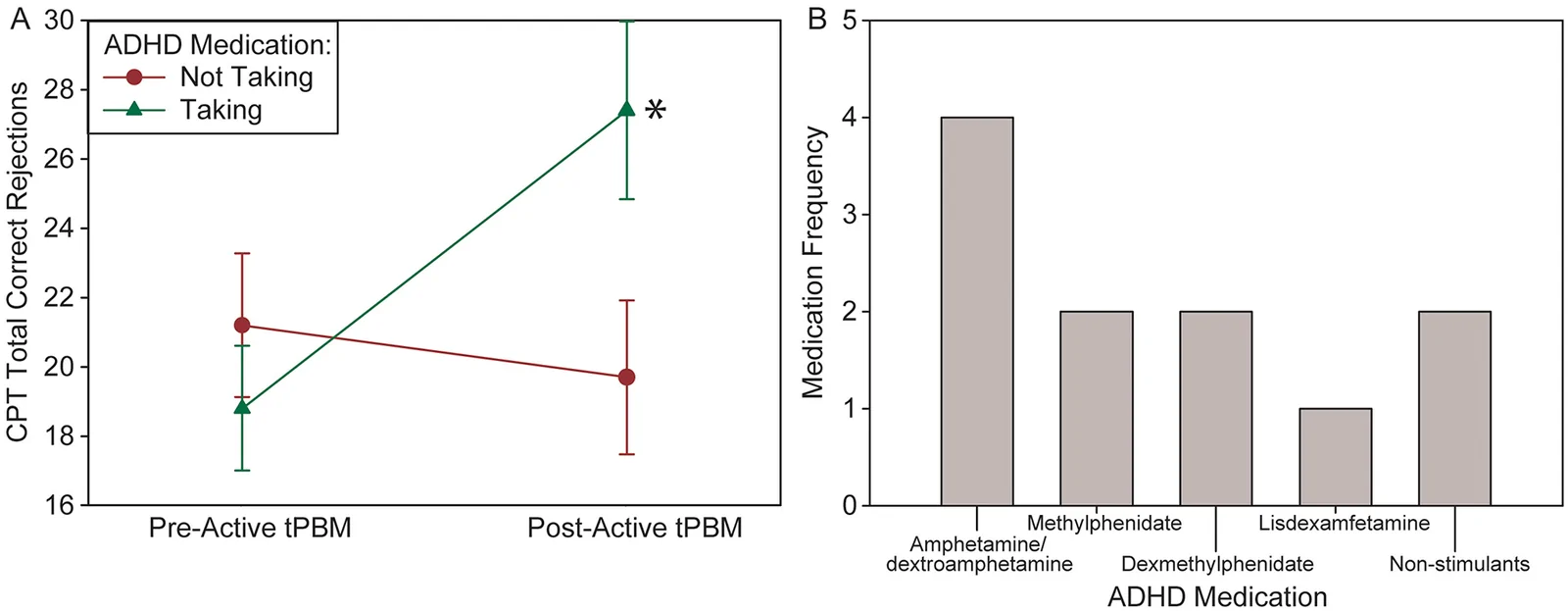
Interaction between ADHD medication status and tPBM treatment response in the active tPBM group. (**A**) Significant interaction between time (Pre-tPBM vs. Post-tPBM) and medication status on CPT total correct rejections among participants who received active tPBM (*p* = 0.002, η²*p* = 0.370). Participants taking ADHD medication showed a marked improvement in performance following tPBM, while those not taking medication showed no significant change. Data are presented as mean ± SEM. ^*^p < 0.05. (**B**) Frequency distribution of specific types of ADHD medications prescribed to participants in the active tPBM group. The non-stimulant category includes two participants, both of whom were prescribed atomoxetine

To further explore whether ADHD medication status influenced changes in CPT performance following active tPBM, a repeated-measures ANOVA was conducted within the active treatment group (*n* = 24), with phase as the within-subjects factor and medication status (on medication: *n* = 10; not on medication: *n* = 14) as the between-subjects factor. The analysis revealed a significant phase × medication interaction on total correct rejections (F_(1,22)_ = 12.94, *p* = 0.002, η²*p* = 0.370). These findings suggest that changes in CPT performance may vary as a function of medication status; however, given the exploratory nature of this analysis and the small, non-randomized subgroup sizes, the results should be interpreted with caution (Fig. 8).

#### Non-significant interaction of Pre-/Post-tPBM phase, tPBM treatment, and ADHD medication status on total hits in the CPT

A three-way ANOVA revealed a non-significant interaction effect between phase, tPBM treatment, and ADHD medication status on total hits (F_(1,45)_ = 2.107, *p* = 0.154). Additionally, there were no significant main effects for tPBM treatment (F_(1,45)_ = 0.711, *p* = 0.404) or ADHD medication status **(**F_(1,45)_ = 0.524, *p* = 0.473). The mean total hits for participants receiving active tPBM was 322.0 ± 7.51 (mean ± SEM), while the sham group averaged 321.0 ± 7.66. These non-significant findings suggest that neither tPBM treatment nor ADHD medication status influenced total hits across phases. The close mean values indicate that all participants were actively engaged in the CPT, regardless of treatment or medication status.

#### Significant effect of tPBM treatment on total correct rejections in 2-back task

**Fig. 9:**
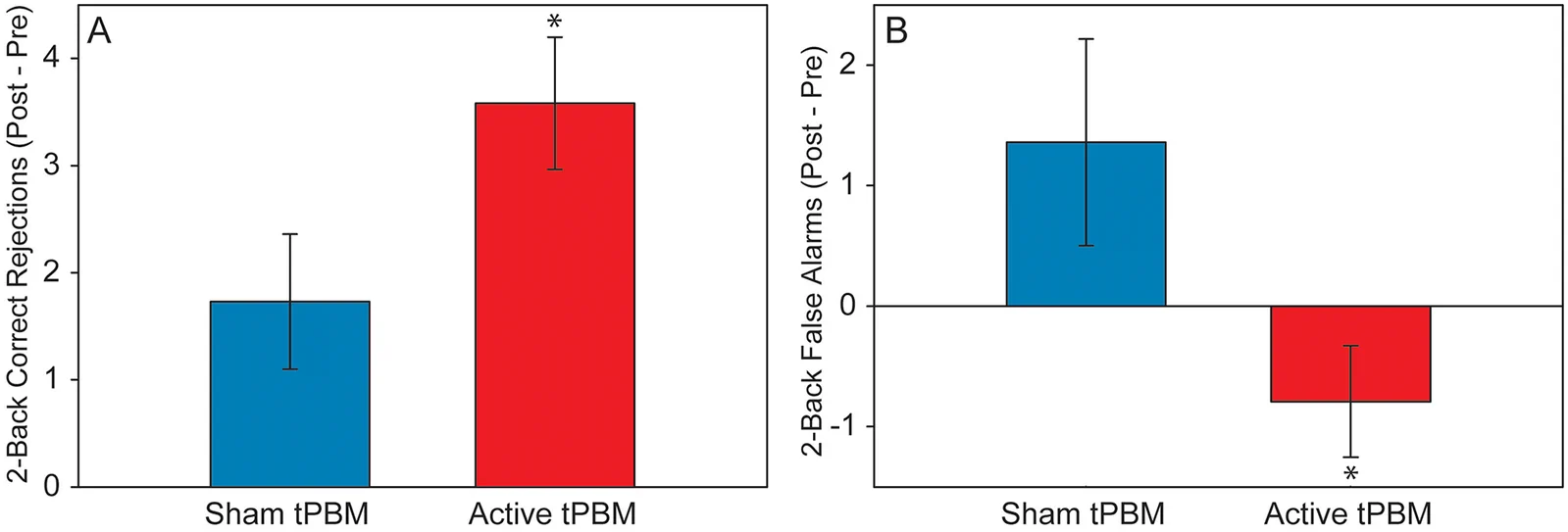
Effects of tPBM treatment on 2-back task performance. (**A**) Participants in the active tPBM group showed a significantly greater increase in correct rejections from pre- to post-treatment compared to the sham group (*p* < 0.05). (**B**) Total false alarms significantly decreased in the active tPBM group compared to the sham group, as assessed by a Mann-Whitney U test (U = 203, *p* = 0.028, rank-biserial correlation = 0.349). Y-axes represent difference scores in these dependent variables (Post-tPBM minus Pre-tPBM). Data are presented as mean ± SEM. ^*^p < 0.05

The normality of total correct rejections in the 2-back task was assessed using the Shapiro-Wilk test. While pre-tPBM scores were normally distributed (*p* > 0.05), post-tPBM scores significantly deviated from normality (*p* < 0.05). Due to this deviation, the non-parametric Mann-Whitney U test was used to compare changes in total correct rejection scores between the sham and active tPBM groups. The analysis revealed a significant difference between groups (U = 207, *p* = 0.04, Rank-Biserial correlation = 0.338), indicating a moderate effect size. The mean change in total correct rejections was 1.73 ± 0.631 for the sham group and 3.58 ± 0.617 for the active tPBM group, demonstrating that tPBM led to a greater improvement in participants’ ability to accurately withhold responses to non-targets compared to the sham group (Fig. 9A).

#### Significant effect of tPBM treatment on total false alarms in the 2-back task

The Shapiro-Wilk test indicated that total false alarms were not normally distributed for both pre-tPBM and post-tPBM data in either the active or sham tPBM groups (*p* < 0.001 for all measures). Consequently, the non-parametric Mann-Whitney U test was used to compare changes in total false alarm scores between the sham and active tPBM groups. The analysis revealed a significant difference between groups (U = 203, *p* = 0.028, Rank-Biserial correlation = 0.349), suggesting a moderate effect size. The mean change in total false alarms was 1.38 ± 0.858 for the sham group and − 0.792 ± 0.462 for the tPBM group, demonstrating that participants in the active tPBM group exhibited greater improvements in response inhibition compared to those in the sham group (Fig. 9B).

#### Significant prefrontal oxygenation improvement following single session tPBM

**Fig. 10:**
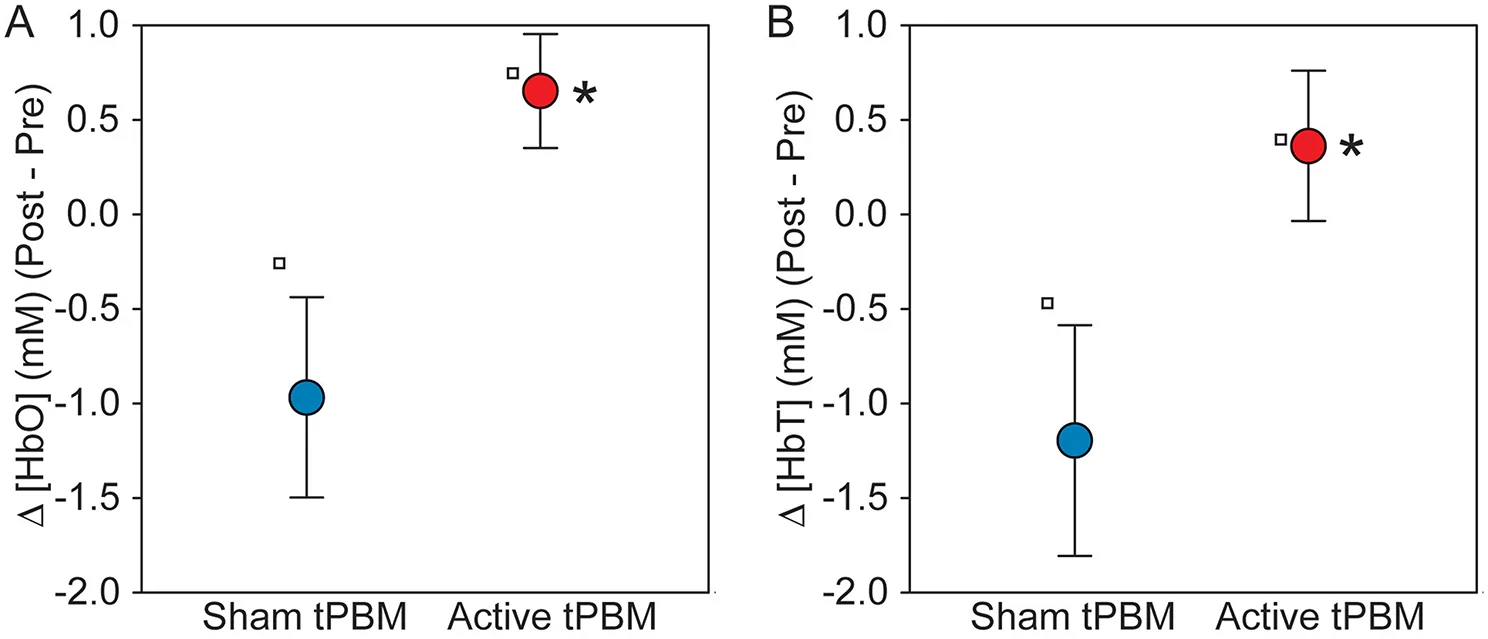
Changes in Δ[HbO] and Δ[HbT] concentrations (µM; averaged across all channels) from pre-tPBM to post-tPBM, measured during a 10-minute fNIRS session. (**A**) A significant group difference was observed in Δ[HbO] concentrations from pre- to post-tPBM, with the active tPBM group showing a significant increase and the sham group showing a decrease (*p* = 0.011, Cohen’s *d* = 0.73). (**B**) Δ[HbT] concentrations also significantly differed between groups, with higher post-treatment levels in the active tPBM group (*p* = 0.038, Cohen’s *d* = 0.59). Data are presented as mean ± SEM; small squares represent the medians. ^*^p < 0.05

Averaged differences in Δ[HbO] and Δ[HbT] concentrations from pre-tPBM to post-tPBM across all 48 fNIRS channels covering the PFC, captured global prefrontal hemodynamic changes during the 10-minute recording sessions. Welch’s t-test showed that participants in the active tPBM group exhibited a significant increase in Δ[HbO], whereas the sham group showed a decrease, resulting in a statistically significant group difference (t_(40.9)_ = -2.67, *p* = 0.011), with a large effect size (Cohen’s *d* = 0.73) (Fig. 10A). Welch’s t-test also revealed a significant group difference in Δ[HbT] concentrations (*t*_(44.2)_ = -2.14, *p* = 0.038), with a moderate-to-large effect size (Cohen’s *d* = 0.59), with higher Δ[HbT] levels observed in the active tPBM group compared to the sham group (Fig. 10B).

## Discussion

### Effects of tPBM on impulsivity in adults with ADHD compared with non-ADHD participants

Results from the initial stage of the study showed that a single session of tPBM to the right forehead improved CPT performance, which relies on sustained attention and impulse control, in individuals with a self-reported medical history of ADHD compared to those who received sham treatment. These findings are consistent with prior evidence from a small case series involving six adults and two children with ADHD, in which red and near-infrared light tPBM applied to four prefrontal sites led to improvements in attention span, impulsivity, and executive functioning [37]. More recently, Zhao et al. in a randomized, sham-controlled study in children with ADHD described that tPBM to the right PFC significantly enhanced selective attention, as reflected by faster reaction times, increased N2pc amplitude, improved attentional tuning, and reductions in inattentive symptoms [29]. However, caution is necessary because their article is a preprint that has not been peer-reviewed. Preprints have not been finalized by authors, might contain errors, and report information that has not yet been endorsed by the scientific or medical community. Although their study focused on pediatric populations and selective attention rather than inhibitory control, the findings provide converging evidence that tPBM over the right PFC modulates core attentional control systems in ADHD. Nevertheless, stimulation parameters differed between their study and the present study. Zhao et al. [29] employed an irradiance of 100 mW/cm² for 8 min, resulting in a fluence of 48 J/cm² and a total energy of 640 J delivered to a single site centered at Fp2. In contrast, the present study applied an irradiance of 250 mW/cm² for 4 min at each site (medial and lateral to Fp2), resulting in a fluence of 60 J/cm², an energy delivery of 816 J per site, and a total energy of 1632 J across both sites. These differences likely reflect anatomical and dosimetry considerations across age groups and should be considered in future research. Overall, these findings support the presence of a shared right prefrontal cognitive control mechanism in ADHD, whereby tPBM may enhance top-down regulatory processes, influencing both attentional allocation and inhibitory control through modulation of prefrontal cortical activity [38].

Meta-analyses of tPBM studies in human participants have shown that tPBM significantly improves attentional performance, with minimal heterogeneity across studies [39]. The studies included in this meta-analysis that involved attention-related tasks consistently applied stimulation over the right PFC, either unilaterally at Fp2 or at medial and lateral sites surrounding Fp2 [12, 15, 40–42]. This consistent targeting suggests that the right PFC may play a preferential role in mediating tPBM-induced improvements in attention, thereby providing a rationale for the unilateral stimulation site used in the current study. Moreover, right, but not left, prefrontal tPBM has been shown to enhance attention-related processes such as attention bias modification in individuals with depressive symptoms [14]. Several possible mechanisms may underlie this lateralized effect of tPBM. The right PFC has more extensive outgoing connections to other brain regions and is more strongly involved in higher-order cognitive processes, including sustained attention and response inhibition. Neuroimaging studies have shown that the right frontopolar regions are preferentially activated during sustained attention tasks [43]. Hypoactivation of the right PFC is a well-established finding in ADHD research, with numerous studies consistently demonstrating reduced activation in this region during cognitive control tasks [44, 45], particularly in the right lateral PFC during tasks requiring response inhibition. Together with results from other studies [15, 16], the findings underscore the importance of targeting the right PFC for improving attention and impulse control in individuals with ADHD.

The first investigation revealed no significant difference in performance between medicated and non-medicated participants. However, the limited ADHD sample sizes in both the medicated group (*n* = 8 for active tPBM; *n* = 5 for sham tPBM) and the non-medicated group (*n* = 7 for active tPBM; *n* = 7 for sham tPBM) may have reduced the statistical power of this first analysis. Of the 223 participants recruited for the initial stage of the study, only 27 reported a history of medically diagnosed ADHD, while the remaining 196 did not. Although the distribution between ADHD and non-ADHD groups was uneven, this first investigation enabled the investigators to initially examine whether ADHD diagnosis moderated the effects of tPBM on impulsivity and sustained attention, as measured by the CPT. The inclusion of a large non-ADHD sample provided a robust control comparison, allowing the investigators to identify diagnosis-specific effects while maintaining the statistical power necessary to detect treatment-related differences. Given the exploratory nature of the first stage of the study, this approach was essential for establishing baseline effects and informing the design of the second stage of the study, which focused on tPBM treatment exclusively in clinical ADHD populations.

To assess the impact of tPBM under stricter ADHD diagnostic criteria, a dual-criteria approach was used, requiring both a self-reported medical diagnosis and elevated symptom severity on the ASRS. This approach required participants to not only report ADHD symptoms that impaired functioning within the past six months but also to have experienced functional deficits significant enough to prompt seeking a psychological or psychiatric diagnosis. Applying this stricter classification excluded three participants from the original sample of 27 individuals with a history of medically diagnosed ADHD. The results showed that the interaction between tPBM treatment and ADHD diagnosis under a dual-criteria approach yielded significance, suggesting that using stricter diagnostic criteria also revealed the effect of ADHD status on the response to tPBM treatment [46].

Regression analyses revealed that ASRS scores were significantly and inversely correlated with total correct rejections on the CPT, indicating that higher ASRS scores predicted poorer CPT performance and greater impulsivity (*r* = -0.214). Using the ASRS to quantify ADHD symptomatology allowed the investigators to assess the strength of the relationship between self-reported symptoms and deficits in impulse and attentional control on the CPT. Supporting these findings, Stern et al. also showed that higher ADHD symptom severity, as measured by ASRS scores, was significantly associated with poorer executive function performance in adults with ADHD [47].

### Cognitive tPBM effects and PFC hemodynamics in adults with ADHD

Given the limited number of adults with a medically diagnosed history of ADHD in the initial stage of the study, the second stage of the study was focused exclusively on recruiting adults who reported a medical diagnosis of ADHD. The investigators anticipated that increasing the sample size within this population would facilitate greater statistical power in detecting treatment effects and allow for better control of potential confounding variables.

Based on prior work demonstrating that tPBM augments PFC metabolic activity leading to improvements in PFC-regulated attention, memory, and executive functions [12, 14, 42, 48, 49], the investigators hypothesized that tPBM would enhance PFC oxygenation, resulting in improved sustained attention and reduced impulsivity in adults with ADHD. Therefore, the second stage of the study investigated underlying hemodynamic mechanisms responsible for the behavioral improvements, potentially linked to enhanced brain oxygenation. It also provided an opportunity to assess whether the behavioral effects observed in the first stage of the study would replicate in a more clinically homogeneous adult ADHD sample, while benefiting from a pre- and post-CPT assessment framework. This allowed for more precise detection of within-subjects changes, increasing sensitivity to the acute effects of tPBM on impulse control and attentional performance.

Exploratory analyses examining the potential moderating role of ADHD medication status suggested that improvements in CPT performance following active tPBM were primarily observed in individuals receiving ADHD medications. These findings should be interpreted with caution, as the study was not specifically powered or designed to detect interaction effects. Medication status was not randomized or incorporated into group assignment, and subgroup sizes were modest. Therefore, this observation should be considered preliminary and requires replication in future studies. One possibility is that ADHD medication may prime the brain, making it more receptive to tPBM, thereby enhancing its neuromodulatory effects. This heightened receptivity could account for the enhanced CPT performance observed in individuals who were on medication and received active tPBM. This interpretation aligns with findings from a recent study involving individuals with bipolar disorder, where taking antipsychotic medication was associated with greater cognitive improvement following tPBM, suggesting a synergistic interaction between pharmacological treatment and tPBM [13].

The significant improvements in total correct rejections and reductions in false alarms on the visual 2-back task in the second stage suggest that tPBM may enhance cognitive control processes in adults with ADHD, consistent with prior findings [30]. Participants in the sham group exhibited higher false alarm rates, reflecting a greater reliance on impulsive responses or difficulties inhibiting responses to non-target stimuli. This may indicate challenges in working memory control, one of the core characteristics of ADHD [50], which has been linked to dysfunction in the frontopolar PFC [51], dlPFC [52], and vlPFC [53]. In contrast, the reduction in false alarms observed in the active tPBM group suggested that tPBM positively influenced impulsivity control, allowing participants to engage in more accurate response inhibition. By decreasing impulsive responses, tPBM likely enhanced participants’ capacity to correctly identify non-targets, resulting in a higher rate of correct rejections. These improvements in both response inhibition (as indicated by decreased false alarms) and target discrimination (as indicated by increased correct rejections) among participants receiving active tPBM reflected enhanced task-specific selective attention and response accuracy compared to the sham group.

Results from fNIRS also showed that participants with ADHD who received active tPBM exhibited a significant increase in both Δ[HbO] and Δ[HbT] concentrations across the entire PFC from pre- to post-tPBM. This change reflects the overall prefrontal hemodynamic response captured by fNIRS channels covering the frontopolar, dorsolateral, ventrolateral, and orbitofrontal PFC during the 10-minute recording period. Similar improvements in PFC hemodynamic response following the same 1064-nm laser tPBM have been demonstrated in previous studies with healthy adults [16, 23, 54, 55].

However, the investigators observed a relative decrease in Δ[HbO] and Δ[HbT] concentrations among sham-treated ADHD participants. Several interpretations may explain this effect. One possibility is that the reduction in task-evoked hemodynamic response reflects ADHD-related deficits in sustaining PFC activation during prolonged cognitive challenges. Individuals with ADHD often exhibit diminished neural recruitment during working memory tasks, especially over time or under conditions of cognitive fatigue [56]. In addition to ADHD-related challenges in sustaining PFC activation, procedure-related cognitive fatigue on Day 2 may have further contributed to the observed reduction in task-evoked hemodynamic response, as the post-fNIRS session followed approximately 40 min of consecutive cognitively demanding activities (pre- and post-CPT, tPBM/sham, and setup time). Without the cognitive enhancement effects of active tPBM, sham-treated ADHD participants may have experienced greater cognitive fatigue or disengagement, contributing to the observed decline in Δ[HbO] and Δ[HbT] levels. This interpretation aligns with previous neuroergonomic research showing that mental fatigue alone can lead to reduced PFC oxygenation during subsequent tasks, even in the absence of physical fatigue [57]. These findings suggest that diminished neural metabolic capacity may compromise PFC hemodynamic activity under prolonged cognitive load, which is consistent with the reduced hemodynamic changes observed in the sham-treated ADHD group. Additionally, motivational deficits and novelty-seeking behaviors observed in individuals with ADHD may have led to reduced engagement with the repeated 2-back working memory task on Day 2, resulting in a blunted prefrontal hemodynamic response [58].

A study in healthy adults showed that PFC hemodynamic responses (Δ[HbO] and Δ[HbT]) remained elevated for more than 10 min of cognitive processing during sustained attention and working memory tasks, following an 8-minute session of tPBM applied to the right forehead [17]. The post-fNIRS recording session started ~ 19 min after the completion of the 8-minute tPBM session. This interval included approximately 1 min to set up the CPT, 14 min to complete the CPT task, and about 4 min for preparing the post-fNIRS and 2-back task simultaneously. All cognitive tasks, the tPBM procedure, and fNIRS recordings were conducted in the same experimental room to minimize time gaps and ensure procedural consistency. Despite the ~ 19-minute delay and the high cognitive demands of the CPT task, particularly in the ADHD population, participants in the active tPBM group still exhibited elevated prefrontal Δ[HbO] and Δ[HbT] during the 10-minute post-fNIRS session. Previous findings suggest that the metabolic effects of tPBM are robust and potentially long-lasting. For example, a single session tPBM has been shown to have a long-lasting effect on CCO activity in the rat brain, as measured after one day, two weeks, and four weeks post-tPBM [59]. However, although prior evidence supports sustained effects beyond 10 min, the ~ 19-minute delay limits the ability to determine the precise duration and decay trajectory of the hemodynamic response in the present study. Therefore, interpreting the observed effects as “sustained” should be done with caution, and future studies are needed to more accurately characterize the temporal profile of tPBM-induced hemodynamic changes in adults with ADHD.

There is an ongoing debate in the field of tPBM regarding the optimal wavelengths to be used in human studies. Light penetration depth is inversely related to both scattering and absorption. Wavelengths with lower scattering and moderate absorption tend to penetrate more deeply. The 1064-nm wavelength offers significantly reduced scattering compared to shorter wavelengths such as 800–850 nm, in accordance with Mie theory [60], and although water absorption is slightly higher at 1064 nm than at 800–850 nm, the overall tissue penetration is superior. This has been confirmed in comparative studies showing that 1064-nm penetrates more deeply than 810 nm and 970 nm [61], and exhibits negligible melanin absorption and reflection relative to wavelengths below 1000 nm [62]. Although 1064 nm is not at the primary absorption peaks of CCO, it benefits from markedly lower scattering. This enables greater photon flux through deeper tissue volumes, potentially leading to greater overall CCO activation compared to shorter wavelengths [63]. Recent neurobehavioral evidence supports this interpretation, showing that tPBM at 1064 nm, applied to the right PFC of healthy participants, enhances working memory capacity more effectively than the 852 nm wavelength [64].

Furthermore, no stimulation-related adverse events or clinically significant side effects were observed following 1064 nm tPBM in the present sample of adults with ADHD. These findings are consistent with recent evidence from healthy adults demonstrating that 1064 nm tPBM is well tolerated and does not produce detectable structural brain abnormalities, neuronal injury, adverse EEG changes, or impairments in executive functioning, further supporting the safety and tolerability of this wavelength in human tPBM applications [65].

### Limitations

First, a technical challenge the investigators encountered is the difficulty of integrating tPBM delivery with simultaneous fNIRS data acquisition. The fNIRS headset covers the forehead, making it impossible to administer laser stimulation and collect hemodynamic signals at the same time. However, real-time monitoring of hemodynamic responses is critical for understanding the immediate effects of tPBM stimulation. Future studies may benefit from using broadband near-infrared spectroscopy (bbNIRS), which enables concurrent tPBM application and real-time data collection without spatial interference between the two systems [16, 55]. Second, during the first stage of the study, blinding efficacy was evaluated in only 27% of the participants, which may constrain the generalizability of these results. Third, although sex was included as a covariate and did not significantly affect the primary outcomes in either stage 1 or stage 2, the unequal sex distribution (70% female [155/223] in stage 1 and 67% female [35/52] in stage 2) may limit the generalizability of the findings. Future studies should aim to recruit more balanced samples to more robustly assess potential sex × treatment interactions. Fourth, the broader age range in stage 2 may have introduced additional variability and potential age-related effects; future studies should consider more restricted age ranges or age-stratified designs. Fifth, ADHD diagnosis was based on self-reported prior clinical diagnosis combined with ASRS screening, without structured clinical interviews or independent verification, which may introduce misclassification bias and limit diagnostic specificity. Finally, this study administered a single session of tPBM to investigate acute effects on both behavioral outcomes and prefrontal hemodynamic responses, but it did not include short-term follow-up assessments. Although prior work has suggested that the hemodynamic effects of a single tPBM session may persist for up to five days in healthy individuals [66], these findings have not been established in adults with ADHD. Future studies should incorporate longitudinal and repeated-session designs to evaluate the durability of effects and inform clinical applications.

## Conclusions

Findings from this single-blind, randomized, sham-controlled investigation suggest that a single session of tPBM applied to the right prefrontal region may be associated with improvements in impulsivity and attentional control. Exploratory analyses further indicated that improvements in impulse control following active tPBM may differ as a function of ADHD medication status, with improvements primarily observed in individuals receiving stimulant or non-stimulant medications. However, these observations should be interpreted with caution, as the study was not designed or powered to test interaction effects, and medication status and class were not randomized. Findings during the 2-back working memory task further support that targeting the right PFC may address cognitive deficits in adults with ADHD. Findings based on a single tPBM session, reflecting acute cognitive and hemodynamic effects, provide a foundation for future longitudinal studies aimed at evaluating whether repeated tPBM sessions can produce sustained clinical improvements in ADHD. Importantly, no adverse events or side effects were reported by participants.

## Supplementary Information

Below is the link to the electronic supplementary material.

Supplementary Material 1

Supplementary Material 2

## Data Availability

The raw data supporting the conclusions of this article will be made available by the corresponding authors upon reasonable request.

## Abbreviations

ADHD: Attention-deficit/hyperactivity disorder
ASRS: Adult ADHD Self-Report Scale
CCO: Cytochrome c oxidase
CPT: Continuous performance task
dlPFC: dorsolateral PFC
DSM-5-TR: The Diagnostic and Statistical Manual of Mental Disorders, Fifth Edition, Text Revision
FDA: Food and Drug Administration
fNIRS: Functional near-infrared spectroscopy
NIR: Near-infrared
PBM: Photobiomodulation
PEBL: Psychology Experiment Building Language
PFC: Prefrontal cortex
SSRIs: Selective serotonin reuptake inhibitors
tPBM: Transcranial photobiomodulation
vlPFC: Ventrolateral PFC
