# Supplementary material for: Transcranial photobiomodulation improves prefrontal oxygenation and impulse control in adults with ADHD: a randomized controlled trial
Source: BMC Psychiatry. 2026 Jul 14;26:767. doi: 10.1186/s12888-026-08391-5 (PMC13651166; doi:10.1186/s12888-026-08391-5)
Supplement: Supplementary file 1 [file 12888_2026_8391_MOESM1_ESM.docx]

Table S1. A complete list of individual medications reported by participants in *Stage 1* of the study.

| *Stage 1* | Number of participants |
| --- | --- |
| **Selective serotonin reuptake inhibitor (SSRI) antidepressants** |  |
| *Sertraline* | 11 |
| *Escitalopram* | 8 |
| *Fluoxetine* | 7 |
| *Fluvoxamine* | 1 |
| **Stimulants** |  |
| *Methylphenidate* | 9 |
| *Amphetamine-dextroamphetamine* | 5 |
| *Dextroamphetamine* | 1 |
| *Lisdexamfetamine* | 1 |
| **Other antidepressants** |  |
| *Bupropion* | 3 |
| *Mirtazapine* | 1 |
| *Duloxetine* | 1 |
| *Venlafaxine* | 1 |
| *Trazodone* | 1 |
| *Amitriptyline* | 1 |
| **Anxiolytics and sedatives** |  |
| *Hydroxyzine* | 4 |
| *Buspirone* | 1 |
| *Propranolol* | 1 |
| *Eszopiclone* | 1 |
| **Mood stabilizers** |  |
| *Lithium carbonate* | 1 |
| *Lamotrigine* | 1 |
| **Opioid antagonist naltrexone** | 1 |
