# Supplementary material for: Transcranial photobiomodulation improves prefrontal oxygenation and impulse control in adults with ADHD: a randomized controlled trial
Source: BMC Psychiatry. 2026 Jul 14;26:767. doi: 10.1186/s12888-026-08391-5 (PMC13651166; doi:10.1186/s12888-026-08391-5)
Supplement: Supplementary file 2 [file 12888_2026_8391_MOESM2_ESM.docx]

Table S2. A complete list of individual medications reported by participants in *Stage 2* of the study.

| *Stage 2* | Number of participants |
| --- | --- |
| ***Stimulants*** |  |
| *Methylphenidate* | 6 |
| *Amphetamine-dextroamphetamine* | 9 |
| *Dextroamphetamine* | 3 |
| *Dexmethylphenidate* | 2 |
| *Lisdexamfetamine* | 2 |
| *Combined amphetamine-dextroamphetamine and lisdexamfetamine* | 1 |
| ***Selective serotonin reuptake inhibitor (SSRI) antidepressants*** |  |
| *Sertraline* | 5 |
| *Escitalopram* | 4 |
| *Fluoxetine* | 1 |
| ***Other antidepressants*** |  |
| *Bupropion* | 1 |
| *Trazodone* | 1 |
| *Desvenlafaxine* | 1 |
| *Vortioxetine* | 1 |
| ***Other psychotropics*** (*alprazolam, clonidine, atomoxetine, olanzapine, propranolol, and hydroxyzine, lamotrigine, zolpidem, quetiapine, pramipexole*) | 18 |
| ***Non-psychotropics*** (*hydrochlorothiazide, albuterol inhaler, omalizumab, pantoprazole, topiramate, levothyroxine, montelukast, atorvastatin, insulin lispro, albuterol sulfate, budesonide-formoterol, spironolactone, azelastine, fluticasone propionate-salmeterol*) | 25 |
